# Potential influences on optimizing long-term musculoskeletal health in children and adolescents with X-linked hypophosphatemia (XLH)

**DOI:** 10.1186/s13023-021-02156-x

**Published:** 2022-01-31

**Authors:** Francis H. Glorieux, Lynda F. Bonewald, Nicholas C. Harvey, Marjolein C. H. van der Meulen

**Affiliations:** 1 Shriners Hospital for Children, McGill University, Montreal, Canada; 2 Indiana Center for Musculoskeletal Health, Indiana University, Indianapolis, IN USA; 3 MRC Lifecourse Epidemiology Centre, University of Southampton, Southampton, UK; 4 Meinig School of Biomedical Engineering, Cornell University, Ithaca, NY USA

**Keywords:** X-linked hypophosphatemia, FGF23, Phosphate homeostasis, Bone mineralization, Musculoskeletal health, Long-term outcomes

## Abstract

In recent years, much progress has been made in understanding the mechanisms of bone growth and development over a lifespan, including the crosstalk between muscle and bone, to achieve optimal structure and function. While there have been significant advances in understanding how to help improve and maintain bone health in normal individuals, there is limited knowledge on whether these mechanisms apply or are compromised in pathological states. X-linked hypophosphatemia (XLH) (ORPHA:89936) is a rare, heritable, renal phosphate-wasting disorder. The resultant chronic hypophosphatemia leads to progressive deterioration in musculoskeletal function, including impaired growth, rickets, and limb deformities in children, as well as lifelong osteomalacia with reduced bone quality and impaired muscle structure and function. The clinical manifestations of the disease vary both in presentation and severity in affected individuals, and many of the consequences of childhood defects persist into adulthood, causing significant morbidity that impacts physical function and quality of life. Intervention to restore phosphate levels early in life during the critical stages of skeletal development in children with XLH could optimize growth and may prevent or reduce bone deformities in childhood. A healthier bone structure, together with improved muscle function, can lead to physical activity enhancing musculoskeletal health throughout life. In adults, continued management may help to maintain the positive effects acquired from childhood treatment, thereby slowing or halting disease progression. In this review, we summarize the opinions from members of a working group with expertise in pediatrics, epidemiology, and bone, joint and muscle biology, on potential outcomes for people with XLH, who have been optimally treated from an early age and continue treatment throughout life.

## Background

X-linked hypophosphatemia (XLH) (ORPHA:89936) is a rare, heritable, progressive, and lifelong disorder caused by increased circulating levels of fibroblast growth factor 23 (FGF23), a phosphate-regulating hormone that leads to reduced renal phosphate reabsorption and consequent abnormal bone mineralization. The resultant chronic hypophosphatemia causes abnormalities in the metabolism of both bone and muscle in association with other systemic defects [1]. The musculoskeletal effects of defective bone and tooth mineralization and growth plate abnormalities usually lead to clinical manifestations in early childhood. The most frequent skeletal presentation in children is rickets, which is associated with slow growth, lower limb deformity and delayed walking with a waddling gait [2]. These manifestations continue into adolescence, with many of the resultant consequences of defects persisting into adulthood, after growth has ceased [3]. Following growth plate closure, the mineralization defects persist in adults with XLH due to years of chronic hypophosphatemia, and they continue to experience significant morbidities, including muscle wasting and osteoarthritis (OA), resulting in a significant reduction in physical function and quality of life [4, 5]. Optimizing bone quality and bone mineralization by maintaining phosphate homeostasis from early childhood is, therefore, important to improve long-term outcomes of people with XLH. Initiation of treatment as early as possible in life is recommended, as this approach will optimize final body height [6].

Recent research has advanced our understanding of skeletal pathology, and the interaction between muscle and bone in healthy individuals, as well as accumulating evidence to elucidate the long-term outcomes of adequate treatment of common musculoskeletal diseases. However, large-scale natural history studies in rare metabolic bone disorders, such as XLH, are distinctly lacking. This presents a challenge to illustrate the importance of optimizing musculoskeletal health in children and adolescents, and the subsequent benefits and improved prognosis of patients in later life.

The current burden of disease observed in adult patients with XLH suggests that delayed/late diagnosis, delayed treatment initiation, and inadequate management during childhood and adolescence may contribute to adverse long-term outcomes in these patients. A thorough understanding of bone and muscle physiology, both from a physical and a metabolic perspective, may provide some answers that could be applied clinically for future management of rare metabolic bone disorders, and to optimize treatment options and outcomes.

This review paper presents the expert views of the authors, based on a series of working group sessions in 2019 that were sponsored and funded by Kyowa Kirin International. The aim of the meetings was to elucidate the consequences of optimizing bone and muscle health in children and adolescents with XLH, and to discuss what impact treatment during various stages of life could potentially have on the prognosis and long-term outcomes of these patients.

The focus of this review is to provide a brief synopsis of XLH and current treatment strategies; provide an academic review of the physical and metabolic aspects of normal bone and muscle, including bone–muscle crosstalk; and discuss the implications of optimal treatment on the long-term musculoskeletal sequelae of XLH, which may also influence other rare metabolic bone disorders.

With an understanding of this information, an expert opinion on the long-term outcomes based on optimizing musculoskeletal health in children and adolescents with XLH is provided. In the context of this review, we also highlight areas for future research to gain a better understanding of the burden of XLH and improve future management.

## Overview of XLH

### Etiology and pathogenesis

XLH is a rare, heritable, X-linked dominant, phosphate-wasting disorder that affects approximately 5 in 100,000 people [7, 8]. XLH is caused by inactivating mutations in the phosphate-regulating endopeptidase homolog on the X chromosome (*PHEX*) gene, leading to enhanced secretion of the phosphaturic hormone FGF23. The ensuing renal phosphate wasting, reduced intestinal phosphate absorption, and low active vitamin D ultimately results in chronic hypophosphatemia [7, 8]. This phosphate insufficiency affects bone mineralization significantly, with low bone turnover and poor bone quality causing rickets in children and osteomalacia in adults [9]. The systemic effects of XLH usually lead to clinical manifestations in early childhood, but the presentation can vary between individuals. The complex musculoskeletal and other system manifestations in childhood include rickets, impaired growth with skeletal deformities, bone pain, muscular dysfunction, craniosynostosis, and dental disorders [7, 10]. In adults, persistent osteomalacia and secondary complications, including early-onset OA, enthesopathies and spinal deformities, can significantly impair quality of life [10, 11]. In addition, many patients may have muscle function deficits, such as muscle weakness, that may be related to insufficient quantities of adenosine triphosphate due to chronic hypophosphatemia [12, 13].

### Progressive and lifelong disease

If the underlying pathophysiological processes leading to the skeletal manifestations of XLH in early childhood are not treated optimally, these defects progress into adolescence and adulthood, resulting in substantial pathology. Once adolescents with XLH enter puberty and the epiphyseal growth plates close, the key radiological features of rickets are no longer evident, despite continued hypophosphatemia throughout life. Although longitudinal bone growth stops after puberty, bone mass continues to be accumulated into early adulthood.

Complete phenotypic rescue in XLH is rarely achieved despite optimal treatment with current strategies. The remaining symptoms can affect multiple systems, demanding continued multidisciplinary specialist care for comprehensive management [10]. Therefore, optimizing bone mineralization and bone quality by maintaining phosphate homeostasis throughout life is the aim to improve long-term outcomes in patients with XLH.

### Treatment of XLH

Familial cases of XLH should be identified within the first months of life based on family history. Early diagnosis is a key challenge in de novo cases of XLH, which occur in approximately 20–30% of patients, with rickets as the most frequent presentation [10, 14]. Immediate initiation of treatment following diagnosis is important and should be commenced as soon as possible to improve long-term outcomes [6, 15].

A consensus statement with the clinical practice recommendations for the diagnosis and management of XLH was published in 2019 [8]. For more than 40 years, children have been treated with multiple daily doses of oral phosphate combined with vitamin D analogs, such as calcitriol and alfacalcidol (conventional therapy), with the aim of improving bone mineralization and healing rickets to correct bone deformities and maximize growth [3, 16–18]. Conventional treatment administration has not been standardized and is variable in both dose and frequency depending on the practicing clinician [19]. This therapy does not aim to maintain normal phosphate levels due to the short half-life of phosphate and the risk of inducing hyperparathyroidism. Moreover, the regimen is burdensome for both patients and families [16, 18]. Conventional therapy may improve rickets, but less so osteomalacia, and has variable effects on skeletal growth. Despite optimal oral therapy with conventional therapy in children with XLH, growth in affected children is often still compromised, which impacts the quality of life in both children and adults [5, 20]. A recent study of 38 children with XLH demonstrated ongoing evidence of radiological and biochemical disease activity on conventional therapy [21].

In adolescents and young adults following growth plate closure, treatment with phosphate supplements and active vitamin D analogs is often stopped, because subjective and skeletal benefits are thought to be lacking and the psychological burden increases, which contributes to poor adherence and lack of follow-up [2, 3, 22]. In adults with XLH, supplementation with oral phosphate and vitamin D analogs is only recommended for symptomatic patients because of limited efficacy related to a slower rate of bone turnover and concerns over safety risks, despite persistence of hypophosphatemia for life [3, 8, 18]. Therefore, many adults do not continue treatment; recent surveys indicate that 15–33% of adult patients were not using any form of conventional therapy [4, 5, 23].

More recently, burosumab, a fully human monoclonal antibody against FGF23 targeting renal phosphate reabsorption, was approved for the treatment of XLH in both children and adults in several countries, including USA, Canada, and the EU, with differing conditions of approval [24–26]. Unlike the fluctuating levels of serum phosphate due to the short half-life of oral phosphate and multiple daily dosing of conventional therapy, burosumab can achieve sustained levels of serum phosphate within the normal range using a once or twice monthly injection in adults and children, respectively [27]. Given the importance of serum phosphate in normal skeletal bone growth and development, correction of serum phosphate levels can be anticipated to lead to improvement of bone mineralization defects and reduce the underlying complications of the disease, thereby improving long-term outcomes and quality of life [19, 21]. Clinical trials of burosumab have shown encouraging results in adults and children with XLH [24–26, 28, 29]. However, data are limited on the use of burosumab in treating adolescents and young adults with XLH from the age of 13–17 years old [30]. Additionally, long-term information regarding the use of burosumab in adults who were previously treated in childhood and adolescence is not available [2]. Conclusive recommendations on the use of burosumab in XLH are premature, and further real-world data on the long-term effectiveness and safety will be required for future updates of the clinical guidelines [8, 21].

## Lifelong implications of the deficits of musculoskeletal development and function in XLH

### Growth

One of the major clinical manifestations of XLH in children is impaired growth; abnormalities of skeletal mineralization cause rickets and bone deformities, particularly bowing of the weight-bearing lower limbs. Children born with XLH have decreased growth velocity by 1 year of age, and their growth progressively declines during childhood compared with normal children, which is more evident during periods of rapid growth in toddlers and during puberty [5, 15, 31, 32].

Healthy skeletal growth during early and late childhood is important to optimize bone health in adulthood [33, 34]. The disordered mineralization in XLH is a generalized defect with a large amount of unmineralized bone matrix of both trabecular and cortical bone and is not limited to the surface of the bone. Periosteocytic lesions cause the distinctive mineralization defects within the mineralized bone [19]. It has been suggested that, in XLH, these lesions are not related to the hypophosphatemia but occur as a direct effect of increased FGF23 expression [35]. This may be one of the reasons why conventional therapy does not improve mineralization of the periosteocytic lesions, resulting in sub-optimal treatment outcomes [19].

Commencing treatment with oral phosphate and vitamin D analogs in early infancy in XLH patients, and before significant growth retardation, improves adult height outcomes and decreases disease activity when compared with starting treatment later [6]. Moreover, in a 64-week Phase II study involving children with XLH aged 1 − 4 years, treatment with burosumab prevented early deceleration in linear growth [28]. In the only Phase III head-to-head study comparing the efficacy and safety of switching children with XLH treated with conventional therapy to burosumab versus continuing with oral phosphate supplementation and calcitriol/alfacalcidol, improvements in length and height z scores were demonstrated [25]. No significant changes in these parameters were observed in the group that had received prior conventional therapy for a mean duration of 4.3 years [25]. This result is consistent with previous findings in which, despite optimal treatment with oral phosphate and vitamin D analogs, 25–40% of patients with well-controlled XLH still had slow gains in linear growth [18], suggesting that inhibition of excess FGF23 may be pivotal in normalizing skeletal growth and improving bone metabolism [36].

### Peak bone mass

**Fig. 1:**
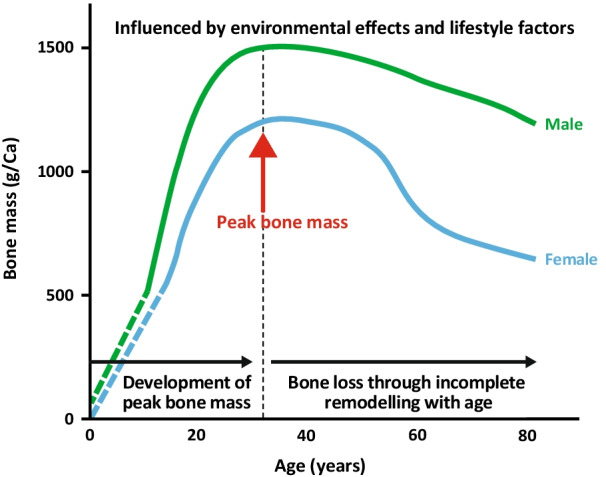
Bone mass across a lifespan. Adapted with permission from Harvey et al. [39]. © 2014 American Society for Bone and Mineral Research

Peak bone mass (PBM) is defined as the maximum amount of bone tissue accrued during an individual’s lifetime once normal growth has ceased and the skeleton has fully matured [37, 38]. The acquisition of bone mass is important for optimal bone health, particularly during the critical childhood growth years and maturation [39, 40] (Fig. 1). Almost half of adult bone mass is attained during adolescence, with 25% being acquired between 12 and 15 years of age [41]. The final PBM is reached in early adulthood, with the exact timing varying by skeletal site and gender. Bone mass is influenced by environmental effects during early development, which may lead to changes that persist into adulthood [42, 43]; lifestyle factors during growth may also influence PBM [38].

The influence of XLH on adult skeletal mass is not completely characterized and studies report variable results. In a study of 19 adults with XLH aged 20–66 years, most of whom had not received medical treatment since puberty, axial (trabecular) bone mass tended to be increased, whereas the peripheral (cortical) bone mass was decreased [44]. Despite these trends, most untreated adults with XLH have normal indices of bone mass. Studies using dual-energy X-ray absorptiometry (DXA) in patients with XLH also suggest that bone mineral density (BMD) is increased at the spine, but not at cortical sites [45]. DXA is a two-dimensional measurement of a three-dimensional structure that only reflects BMD and does not provide information on compartment-specific BMD and bone quality [46, 47]. Additional calcifications and enthesopathy may also influence these measurements, with resultant artefactual increases, and therefore DXA should be interpreted with caution in patients with XLH. The DXA findings are also supported by studies in patients with XLH using peripheral quantitative computed tomography (pQCT), which can be used to assess bone geometry, microarchitecture, and compartment-specific volumetric bone mineral density (vBMD) [48]. Because cortical vBMD largely reflects the average degree of mineralization of cortical bone tissue, this parameter could be useful to study the skeletal effects of XLH and to assess the consequences of treatment modalities on the changes in bone mineralization. In a study using pQCT, vBMD increased for trabecular bone in the lumbar spine but was somewhat decreased for cortical bone at the radial diaphysis [49]. The lower cortical vBMD likely reflects the underlying mineralization defects that may not be completely corrected by therapy with oral phosphate supplementation and vitamin D analogs [49]. In patients with XLH, these defects of the tissue matrix are thought to create a “vicious cycle”, in which the softer tissue leads to greater strain, increased osteocyte stimulation and increased formation of poor-quality bone, which may explain why these patients may have normal or even increased levels of BMD [47].

Quantitative ultrasound (QUS) is a convenient non-invasive, cost-effective, and radiation-free technology that may be utilized for bone mass and quality assessment in the future. Studies in adults confirm that the information provided by QUS measurements is partly independent of BMD. More recently, the bidirectional axial transmission modality of QUS has been used to elucidate changes in cortical bone properties that contribute to bone stiffness and strength [47]. In a recent pilot study in children with XLH using this modality, a reduction in mechanical competence of cortical bone and in the clinical phenotype was shown, despite all patients being on conventional treatment [47]. As a regular complementary radiation-free monitoring tool in children, QUS may provide valuable information on bone quality to optimize treatment of patients with XLH in the future.

### Whole bone mechanical properties

Whole bone morphology is an important determinant of skeletal mechanics during locomotion and in applied loading models [50–52]. Any developmental changes in bone size and shape can alter the induced stresses and strains on the skeleton during loading [53]. Whole bone mechanical properties are influenced by structural and geometric features, such as cortical thickness, spatial distribution of trabecular bone, cross-sectional area (CSA), and bone size and shape, as well as by changes that occur with ageing and disease [54]. Mechanical loading, in addition to genetic, environmental, and nutritional factors, plays an important role in determining bone strength, which is critical for optimal bone health and quality of life [54, 55].

**Fig. 2:**
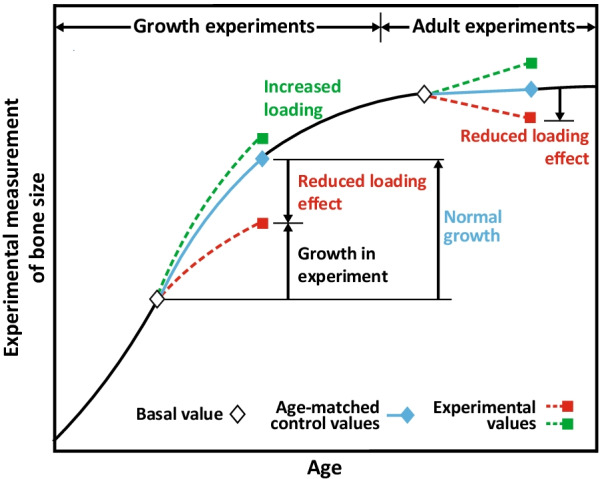
Effects of mechanical loading on bone size in murine models in vivo*.* Adapted with permission from van der Meulen et al. [58]. © 1995 Orthopaedic Research Society

In the case of low or reduced levels of loading, bone strength decreases at rates of up to 2.5% per month, whereas regular exercise leads to increases in bone strength [56]. In children, the effects of physical activity can be seen in a relatively short period. Prepubescent children randomized to jumping exercises 3 days a week had significantly increased bone mineral content in the femoral neck and lumbar spine after only 7 months compared with those randomized to stretching exercises [57]. Figure 2 summarizes data across many adaptation experiments in murine models to illustrate that altered mechanical loading during skeletal development produces concomitant changes in bone size [58], which has also been seen in human subjects [55, 59]. These differences persist during ageing. The greatest effects of alterations of loading on bone are seen during growth. When loading is increased or decreased after skeletal maturity, the effects are smaller and are primarily seen in the endosteum, not the periosteal envelope [55, 59].

These features of mechanical loading are particularly relevant to growth in XLH, in which muscle structure and function are compromised, thereby reducing the load applied to the skeleton during development [7, 13]. In a study of 34 patients with XLH, muscle density and volume were lower and associated with lower bodyweight-related peak muscle force and power when compared with age- and gender-matched controls [13]. Patients with XLH and no lower-leg deformities demonstrated better muscle function than those with severe deformities but, in both cases, this function was still significantly below that of controls, suggesting that limb deformity is only one of several contributing factors to reduced muscle function in XLH [13]. Additionally, in a cohort of 21 adults with XLH, muscle strength, power and aerobic fitness were impaired, and lower body function appeared to be more affected than other components of physical function [60]. The muscle function deficit in XLH would be expected to lead to weaker bones in these individuals because muscle force is strongly correlated to bone strength in healthy subjects [61]. However, bone mass and size at the distal tibia were increased in a study of 30 patients with XLH, when compared with controls. A higher bone mineral content in the distal tibia, even with decreased muscle power, was mostly explained by a higher bone CSA [61]. This increase in bone was accompanied by a deficit in muscle function, despite muscle CSA being unaffected, suggesting that muscle tissue material properties could be compromised [50]. Therefore, bone mechanosensing may be compromised and bone–muscle interactions are likely altered as discussed below.

Mechanosensing osteocytes are involved in the regulation of bone mass in XLH, and the distinctive periosteocytic lesions may have functional implications for bone homeostasis [19, 62]. A hypomineralized matrix and greater bone deformation in XLH would lead to disturbed mechanosensing by the osteocytes with loading. This is a result of the bones being softer because of the undermineralization of the osteocyte perilacunar matrix, which may contribute to the higher trabecular bone mass found in patients with XLH [19]. In theory, the higher strain would induce osteocytes to send signals of bone formation, but the bone produced is hypomineralized due to reduced phosphate leading to a vicious cycle.

### Bone–cartilage crosstalk and OA

Mechanical loading can increase bone mass but also has a significant impact on joints. Joint tissues are sensitive to the mechanical environment, with mechanical loading being one of the most important external factors for cartilage homeostasis and for regulating the development and long-term integrity of the joint [63, 64]. However, abnormal loading may have a negative effect on the joints, with resultant cartilage degradation from both disuse and overuse [63].

OA represents a group of multifactorial joint diseases characterized by degeneration of articular cartilage, synovial inflammation, and changes in periarticular and subchondral bone, and is considered a disease of the entire joint [65, 66]. It is not merely a process of wear and tear, but rather an abnormal remodeling of bone and joint failure, involving both genetic and acquired factors including age, gender, prior joint injury, and mechanical factors such as malalignment and abnormal joint shape [65, 67]. OA is associated with changes to bone mass and/or stiffness of the subchondral bone, altering the stresses that are transmitted to the cartilage.

The start of early OA and the later progression of the disease are thought to be two distinct pathophysiological processes [68]. During the initiation and early-stage OA, the subchondral bone mass is reduced and is associated with lower tissue modulus [66]. Changes in joint shape and load transmission may be caused by the increase in bone remodeling, thereby predisposing to progressive loss in cartilage [66]. In late-stage progression of OA, the subchondral bone densifies and becomes sclerotic without a decrease in bone formation, but the subchondral bone stiffness is low and is accompanied by a decrease in mineralization [68, 69]. The development of OA is also associated with abnormal or misaligned mechanical forces, together with increased bone remodeling [68].

In XLH, adults are affected by early-onset and accelerated OA in the weight-bearing joints. The incidence of OA in this group has been highlighted in two clinical studies. In the Phase III placebo-controlled study using burosumab, the baseline characteristics revealed that 68.7% and 63.4% of participants had a history of orthopedic surgery and OA, respectively [70]. In a second Phase III single-arm study investigating the effects of burosumab on histomorphometric measure of osteomalacia, 57% of subjects had OA at study entry [26].

OA in XLH may be driven by the skeletal biomechanics, which will be affected by bone material properties and bone shape. The extent to which XLH influences the shape of the hip joint is unknown and could be a predictor of the development of OA that may ultimately require arthroplasty. An active shape model that more precisely quantifies the deforming effects of OA on the proximal femur may be used in early stages of the disease to identify individuals who are at the highest risk of developing OA and may require surgery. An active shape model could, therefore, be useful as an imaging biomarker in patients with XLH to assess effects of long-term treatment in hip OA [71].

### Metabolic aspects of muscle–bone interactions

Historically, the nature of muscle–bone crosstalk was believed to be purely mechanical, with mechanical loading being a key mechanism linking both muscle and bone through a central promoting role of physical activity [72]. However, bone and muscle are now also understood to act as secretory endocrine organs, affecting the function of each other [73]. Muscle produces factors that: (1) can have a positive or negative effect on bone depending upon the level of physical activity; (2) enhance the effects of mechanical loading; and (3) delay the effects of ageing [73, 74].

Patients with XLH present with excess FGF23 expression and chronic hypophosphatemia, which mediate many of the clinical manifestations related to both the physical and metabolic effects of the musculoskeletal system [7]. Despite having normal muscle size, muscle density along with peak muscle force and power are reduced in patients with XLH [13]. The muscle deficits in XLH are likely to be caused by phosphate insufficiency rather than the direct effect of high FGF23 levels [75].

The skeletal muscle secretome accounts for various molecules that affect bone, including insulin-like growth factor 1 (IGF-1); basic fibroblast growth factor 2 (FGF2); interleukins (IL-6, IL-8, IL-15); irisin; myostatin; osteoglycin; family with sequence similarity 5 member C (FAM5C); transmembrane protein 119 (Tmem119); and osteoactivin [72, 76].

In addition, β-aminoisobutyric acid (BAIBA) has been identified as an osteocyte survival factor produced by skeletal muscle during exercise that protects against glucocorticoid-induced osteocyte apoptosis [77]. Levorotatory β-aminoisobutyric acid (L-BAIBA) is also a regulator of FGF23 expression in bone and, therefore, may play a role in phosphate metabolism [78, 79].

Even though studies on the potential effects of bone on muscle metabolism are sparse, a few osteokines have been identified. Prostaglandin E2 (PGE2) and Wnt3a are secreted by osteocytes, and osteocalcin and IGF-1 are produced by osteoblasts. Together with sclerostin, which is secreted by osteocytes, these osteokines may have an impact on skeletal muscle cells [72]. In vivo studies show that osteocytes produce factors that decrease muscle mass and function with age, suggesting that during growth and development, osteocytes produce factors that have a positive effect on muscle mass and function, with increased contractile force and size [80].

PHEX and dentin matrix acidic phosphoprotein-1 (DMP-1) are two of a range of biomarkers for osteocyte differentiation. These proteins are expressed in late osteoblasts/early osteoid osteocytes while they are embedding in the osteoid matrix [81, 82]. Matrix extracellular phosphoglycoprotein (MEPE) and FGF23 are expressed later in the maturing osteocyte in mineralizing bone [83, 84]. DMP-1 and PHEX downregulate FGF23 [85]; MEPE increases FGF23 levels indirectly via inhibition of PHEX enzymatic activity [86, 87]. FGF23 is also regulated by serum phosphate levels independent of the actions of calcitriol [84, 88]. In XLH, the loss-of-function mutations of the *PHEX* gene dramatically increase FGF23 production in bone, and the resultant hypophosphatemia causes a decrease in bone mineralization [81, 82]. The presence of hypomineralized periosteocytic lesions that accumulate osteopontin is one of the characteristic hallmarks of XLH [19, 62, 89]. They are never seen in other hypophosphatemic disorders, clearly indicating that the osteocyte is a central target in XLH. Although osteopontin secretion is elevated in hypophosphatemic rickets and acts locally to delay mineralization, no direct link has been made between high FGF23 and high osteopontin levels. Several factors are likely involved in these hypomineralized lesions within the bone extracellular matrix [62, 90].

Enthesis development is also impacted in XLH. Enthesopathy occurs where fibrocartilage mineralizes at tendon insertion points, which can appear in patients with or without phosphate treatment [7]. The effects of FGF23 on fibrocartilage mineralization are unclear and further research is required to understand how soft tissue mineralizes. In XLH, it is not known if the calcification of the fibrocartilage is a compensatory response to weaker bones [7].

## Expert opinion on optimizing musculoskeletal health in XLH by restoring phosphate homeostasis

Limited treatment options are available for rare genetic musculoskeletal disorders such as XLH, and gaps of knowledge persist in natural disease history, long-term risks, disease management, and quality of life in those affected [91]. The limited awareness among healthcare professionals regarding these disorders is compounded by natural variability in clinical presentation and disease burden. Therefore, it may take years for an appropriate diagnosis and for the patient to access quality specialist care. These issues are worsened by limited research funding in rare diseases. The James Lind Alliance (JLA), hosted by the National Institute for Health Research, is a non-profit initiative providing a transparent approach, supervised by an impartial JLA adviser, that brings patients, carers and healthcare professionals together for a research priority setting partnership (PSP) [91]. One of these partnerships applied the JLA method to a group of rare musculoskeletal disorders in adults, including XLH, to identify the most important research directions in diagnosis, treatment, and long-term management. They reported that one of the main overarching knowledge gaps appears to be the need for better understanding of rare metabolic bone disease progression in adulthood [91]. This recommendation, together with the absence of data on long-term outcomes of XLH, led to several workshops with the authors to provide their expert opinion on optimizing musculoskeletal health and in identifying key areas that may affect the long-term prognosis of XLH, based on current research, knowledge of the pathophysiology of the disease, and current treatment options.

In XLH, the persistent hypophosphatemia with resultant clinical manifestations and complications of abnormal musculoskeletal development, play a significant role in the disease burden [5]. If phosphate levels can be restored during the pivotal timeframe of skeletal growth, it is anticipated that musculoskeletal benefits acquired during childhood could persist later in life, particularly if these involve more permanent features such as impaired growth and/or skeletal deformity.

Therefore, the prognosis in later life of optimally treated children with XLH should be significantly improved compared with those who are inadequately treated or undertreated, although this effect may be difficult to quantify over a set time period. Enduring benefits of correcting phosphate homeostasis and improving bone mineralization during childhood will be related to skeletal improvements obtained during the pivotal phase of skeletal growth and maturation:

- Greater treatment efficacy starting as early as possible in children with XLH underpins improved skeletal development and causes growth to peak and may change the long-term growth trajectory.
- Bone geometry observed in patients with XLH reflects adaptation to poor bone tissue properties; associated joint deformities are likely to contribute to the development of precocious OA in adulthood. However, with early and optimal treatment, improved bone mineralization and bone shape during early growth should help to prevent or reduce bone deformities. Avoiding or correcting deformities is expected to result in ameliorating the chronic phenotype of OA, stiffness, and enthesopathy, thereby reducing pain and improving mobility throughout the patient’s lifetime.
- Increased bone mineralization, reduction in limb deformities, and improved muscle function should lead to increased levels of physical activity. The improvement in musculoskeletal health, with a reduction in muscle weakness, may positively impact the long-term quality of life in all patients, with better mobility and a reduction in pain and stiffness further promoting physical activity.

Discontinuation of therapy after growth plate closure will lead to the inevitable reoccurrence of chronic hypophosphatemia. While it is reasonable to predict that patients treated through childhood would have a longer window of improved skeletal health and life quality in adulthood, increasing poor skeletal mineralization, osteomalacia, and the impact on muscle function will lead to a return of clinical manifestations:

- One of the first effects of the return of hypophosphatemia would be the appearance of muscle weakness and fatigue. If phosphate is not corrected, the subsequent extended muscle weakness can affect not only bone health but also overall metabolism. The original muscle phenotype may revert on the cessation of treatment, with decreased physical activity leading to additional adverse effects.
- Enthesopathies may increase, possibly as a result of increases in inflammation.
- Elevated FGF23 and low phosphate levels will inhibit mineralization of newly formed bone matrix in adults, and the softening of the bone matrix may result in abnormal biomechanical loading and shape changes of the lower limbs, even if not as severe as those in untreated individuals. The development of OA is likely to be earlier than in the general population. However, OA will possibly be delayed in adult patients with XLH who were treated optimally earlier on in life.

Based on the discussion above, optimal and early treatment of XLH throughout childhood, ideally seeking to restore phosphate homeostasis and thus correct the hypophosphatemia, should have long-lasting benefits in adulthood. The impact of lifelong treatment on bone mineralization and bone quality should reduce the incidence and severity of musculoskeletal defects, and therefore delay the onset of precocious complications of the disease. In adolescents and adults after growth plate closure, continued treatment should at least maintain or further improve bone mineralization and reduce osteomalacia. This treatment may have a significant impact on the long-term sequelae of the disease and improve overall quality of life during their lifespan.

## Conclusions

In children with XLH, the importance of early intervention and treatment to achieve better outcomes is well established. However, the long-term benefits of optimizing musculoskeletal health in children with XLH have been less well documented. The optimal long-term outcomes of children and adolescents with XLH need to be ensured to improve morbidity, promote good quality of life, and maintain adequate physical function into adulthood.

Further studies to better understand the natural disease history, long-term risks, disease management, and quality of life in those affected by rare metabolic diseases are warranted. While the impact of optimizing treatment with burosumab is not yet known for the long-term outcomes of children with XLH, new areas of research, including pathophysiology of bone, muscle, and joints, as well as crosstalk within the bone–muscle–joint axis, may shed light on what could be achieved in the future.

Appropriate therapy and management to improve phosphate homeostasis early on in life in individuals with XLH may offer the possibility to alter the clinical trajectory, thereby changing the course of the disease. In addition, continuation of treatment that targets the pathophysiology of the disease into adulthood is likely to address the ongoing progressive musculoskeletal disease seen in patients with XLH and maintain the benefits acquired during childhood.

## Data Availability

Data sharing is not applicable to this article as no datasets were generated or analyzed during the current study. Any data referred to in the text are available from the attached references.

## Abbreviations

BAIBA: β-Aminoisobutyric acid
BMD: Bone mineral density
CSA: Cross-sectional area
DMP-1: Dentin matrix acidic phosphoprotein-1
DXA: Dual-energy X-ray absorptiometry
FAM5C: Family with sequence similarity 5 member C
FGF2: Fibroblast growth factor 2
FGF23: Fibroblast growth factor 23
IGF-1: Insulin-like growth factor 1
IL-6/8/15: Interleukin-6/8/15
JLA: James Lind Alliance
L-BAIBA: Levorotatory β-aminoisobutyric acid
MEPE: Matrix extracellular phosphoglycoprotein
OA: Osteoarthritis
PBM: Peak bone mass
PGE2: Prostaglandin E2
PHEX: Phosphate-regulating endopeptidase homolog on the X chromosome
pQCT: Peripheral quantitative computed tomography
PSP: Priority setting partnership
QUS: Quantitative ultrasound
Tmem119: Transmembrane protein 119
vBMD: Volumetric bone mineral density
XLH: X-linked hypophosphatemia
